# Maternal beliefs and asthma medication adherence during pregnancy

**DOI:** 10.1016/j.jacig.2025.100579

**Published:** 2025-10-10

**Authors:** Kelly Colas, Jennifer Namazy, Diana Johnson, Alec L. Todd, Christina D. Chambers

**Affiliations:** a Division of Allergy and Infectious Diseases, University of Washington, Seattle, Wash; b Department of Allergy and Immunology, Scripps Clinic, San Diego, Calif; c Department of Pediatrics, University of California San Diego, La Jolla, Calif

**Keywords:** Pregnancy, asthma, medication adherence, medication belief, infant outcomes

## Abstract

**Background:**

Maintaining optimal control of asthma during pregnancy is critical, as poorly controlled asthma is associated with negative health implications for both mother and baby. However, asthma medications are frequently discontinued by patients during pregnancy.

**Objective:**

This study aimed to examine maternal beliefs, maternal characteristics, and infant birth outcomes associated with asthma medication nonadherence during pregnancy.

**Methods:**

Between 2014 and 2022, pregnant asthmatic women were invited to respond to a 29-question asthma adherence survey. Using the *t* test, chi-square test, or Fisher exact test, we compared maternal characteristics and beliefs about asthma medications between those survey respondents with more reported adherence and those survey respondents with less reported adherence. Additionally, we used logistic regression with adjustment for select covariates to compare birth outcomes between those survey participants with more and those participants with less adherence.

**Results:**

Of the 341 participants who responded to the asthma medication adherence question, 40 (11.7%) reported less adherence. Less adherence was associated with agreement with the statement that taking asthma medications in pregnancy is unsafe (*P* = .001), concern that a preventive/controller medication would harm the baby (*P* = .003), and disagreement with the belief that asthma medication is effective in controlling symptoms in pregnancy (*P* = .021). Less adherence was not associated with preterm delivery (odds ratio [OR] = 0.54 [95% CI = 0.12-2.38]), small for gestational age from the standpoint of birth weight (adjusted OR = 0.31 [95% CI = 0.06-1.76]), or small for gestational age from the standpoint of birth length (adjusted OR = 0.45 [95% CI = 0.05-4.43]).

**Conclusion:**

Although we did not find an association between any maternal characteristics and asthma adherence, concerns about the safety and efficacy of asthma medication were significantly associated with asthma medication adherence.

## Introduction

Asthma is the most common chronic respiratory condition encountered during pregnancy.^1^, ^2^, ^3^ Optimal control of asthma symptoms is critical, as poorly controlled asthma is associated with both adverse effects for mother (including pregnancy and delivery complications) and adverse effects for baby (eg, increased rates of prematurity, small for gestational age [SGA], higher levels of care required in the neonatal intensive care unit, a greater likelihood of developing asthma themselves).^4^^,^^5^

However, up to 40% of patients discontinue their asthma medications during pregnancy.^6^ Factors associated with nonadherence during pregnancy include being an ex-smoker, current cigarette smoking, non-White race/ethnicity, lower lung function, younger maternal age, higher parity, and no prescription of an inhaled corticosteroid (ICS) at baseline.^6^ There is limited research regarding reasons for nonadherence during pregnancy. The aim of this study was to examine participant characteristics, beliefs, and infant outcomes associated with asthma medication nonadherence during pregnancy.

Data were collected by the MotherToBaby Pregnancy Studies, a US and Canada-wide prospective cohort study of pregnancy exposures and outcomes. This parent study has been described in detail previously.^7^ Briefly, pregnant women are enrolled and followed throughout pregnancy and through 1 year postpartum by using maternal interviews and medical record reviews to capture all pregnancy exposures, pregnancy and infant outcomes, and covariates. A 29-question asthma adherence survey was added to the MotherToBaby study protocol in 2014 as an optional online questionnaire for study participants who indicated having a current diagnosis of asthma. The survey was completed at the time of enrollment for this study. In addition, the Asthma Control Test was administered at the time of enrollment as a measure of symptom control in the previous 4 weeks.

On the basis of responses to the statement “I follow my preventive/controller asthma medication plan,” more adherence was defined as a response of 1 (I agree completely), 2 (I agree mostly), or 3 (I agree somewhat). Less adherence was defined as a response of 4 (I disagree somewhat), 5 (I disagree mostly), or 6 (I disagree completely) with this statement. As appropriate, the *t* test, chi-square text, or Fisher exact test were used to compare maternal characteristics and beliefs about asthma medications between those participants with more adherence and those with less adherence.

Maternal beliefs about asthma medication were additionally analyzed by using logistic regression and inverse probability of treatment weighting (IPTW) to estimate crude odds ratios (ORs) and IPTW adjusted odds ratios (aORs) and 95% CIs. IPTW aORs were reported when 1 or more covariates met the inclusion criteria for a confounder, which was defined as a change in the OR greater than 10% when the covariate was included in a logistic regression model predicting the association between maternal beliefs and medication adherence. One exception is the Global Initiative for Asthma assessment of asthma severity, as it was not recorded before medication adherence in pregnancy and therefore may not qualify as a true confounder. The selected covariates were then used to create a propensity score for less adherence The associations between asthma medication adherence and birth outcomes, including SGA (defined as ≤10th percentile for sex and gestational age using standard US growth curves) from the standpoints of birth weight, length, head circumference, and preterm delivery (<37 weeks’ gestation) were analyzed. Birth outcomes were compared by using the same statistical methodology, with logistic regression and IPTW used to estimate crude ORs and IPTW aORs and 95% CIs, including selected covariates that were associated with both the exposure and the outcome. The statistical packages SPSS, version 29.0.2.0, and R, version 4.3.3, were used to perform the analyses. All participants provided informed consent, and the study was approved by the University of California San Diego Human Research Protections Program.

## Results and discussion

A total of 396 participants completed the survey between 2014 and 2022. Of the 396 participants, 341 (86.1%) responded to the question about medication adherence; all further analyses of maternal characteristics and beliefs were restricted to that subset. Maternal characteristics of these 341 participants are described in Table I. Most participants reported being of the White race (83.7%) and having an education level of at least some college completed (94.5%). Of these 341 participants, 319 were pregnant and 10 were postpartum at the time of enrollment. The pregnancy status of the additional 12 participants at the time of enrollment was unknown. For pregnancy and infant outcomes, the sample varied by outcome.

**Table I:** Maternal characteristics

Characteristic	All participants (N = 341)
Age (y), no (%)	
18-34	219 (64.2)
≥35	122 (35.8)
Race, no. (%)∗	
White	283 (83.7)
Asian	22 (6.5)
Black	16 (4.7)
Native American	5 (1.5)
Pacific Islander	1 (0.3)
Other	11 (3.3)
Maternal education, no. (%)	
Some high school (10th or 11th grade)	1 (0.3)
High school graduate (includes trade school)	18 (5.3)
Some college (or specialization)	50 (14.7)
College or university graduate	120 (35.2)
Postcollege graduate	152 (44.6)
Household income, no. (%)†	
<$50, 000	47 (14.2)
≥$50, 000	283 (85.8)
GINA classification, no. (%)‡	
Mild (1-3)	203 (69.3)
Severe (4-5)	90 (30.7)

*GINA*, Global Initiative for Asthma.

∗ Total of 3 subjects with missing values for race.

† Total of 11 subjects with missing values for household income.

‡ Total of 48 subjects with missing values for GINA classification.

Less adherence was reported by 40 participants (11.7%), and better adherence was reported by 301 participants (88.3%). No maternal characteristics differed statistically significantly between less adherent and more adherent participants (Table II).

**Table II:** Maternal characteristics by asthma medication adherence

Characteristic	Less adherence (11.7%) (n = 40)	More adherence (88.3%) (n = 301)	*P* value∗
Maternal age at estimated delivery date (y), (categoric), no. (%)			
≤29	11 (27.5%)	53 (17.6%)	.244
30-34	11 (27.5%)	113 (37.5%)	
>34	18 (45.0%)	135 (44.9%)	
Maternal race, no. (%)†			
White	31 (77.5%)	252 (84.6%)	.364
Non-White	9 (22.5%)	46 (15.4%)	
Maternal ethnicity, no. (%)‡			
Non-Hispanic	37 (92.5%)	272 (90.7%)	1.000
Hispanic	3 (7.5%)	28 (9.3%)	
Maternal prepregnancy BMI (kg/m^2^), no. (%)§			
<18.5 (underweight)	1 (2.6%)	6 (2.0%)	.578
18.5-24.9 (normal weight)	16 (41.0%)	129 (43.4%)	
25-29.9 (overweight)	9 (23.1%)	88 (29.6%)	
≥30 (obese)	13 (33.3%)	74 (24.9%)	
Hollingshead socioeconomic category, no. (%)‖			
Low	1 (2.5%)	12 (4.0%)	1.000
High	39 (97.5%)	288 (96.0%)	
Parity (previous live birth[s] or stillbirth delivery[ies]), no. (%)			
0	25 (62.5%)	157 (52.2%)	.288
>0	15 (37.5%)	144 (47.8%)	
Asthma Control Test score at enrollment <20, no. (%)¶	27 (35.5%)	80 (31.1%)	.561
GINA classification at last menstrual period, no. (%)#			.894
Mild (1-3)	23 (71.9%)	180 (69.0%)	
Severe (4-5)	9 (28.1%)	81 (31.0%)	
Pregestational hypertension, no. (%)	3 (4.5%)	8 (3.6%)	.719
Gestational age at date of survey (continuous), mean (SD)	23.4 (9.4)	21.7 (10.2)	.268
Gestational age at date of survey (categoric), no. (%)			
First trimester (2.0-13.0 weeks' gestation)	4 (10.0%)	71 (23.6%)	.123
Second trimester (13.1-26.0 weeks' gestation)	23 (57.5%)	135 (44.9%)	
Third trimester (>26.0 weeks' gestation) or postpartum	13 (32.5%)	95 (31.6%)	

*BMI*, Body mass index; *GINA,* Global Initiative for Asthma.

∗ Two-sample *t* test for continuous variables, chi-square test (or Fisher exact test when the expected number in the cell is <5) for categoric variables.

† Total of 3 subjects with missing values for maternal race (more adherence).

‡ Total of 1 subject with missing values for maternal ethnicity (more adherence).

§ Total of 1 subject with missing values for maternal prepregnancy BMI indicating less adherence and 4 subjects with missing values for maternal prepregnancy BMI indicating more adherence.

‖ Total of 1 subject with missing values for Hollingshead socioeconomic category indicating more adherence.

¶ Total of 1 subject with missing values for asthma symptoms that were well controlled over the month before intake, indicating less adherence, and 7 subjects with missing values for asthma symptoms that were well controlled over the month before intake, indicating more adherence.

# Total of 8 subjects with missing values for GINA Classification at first day of last menstrual period indicating poor adherence and 40 subjects with missing values for GINA Classification at first day of last menstrual period indicating good adherence.

However, less adherence was associated with agreement with the statement that taking asthma medications in pregnancy is unsafe (*P* = .001), concern that a preventive/controller medication would harm the baby (*P* = .003), and disagreement with the belief that asthma medication is effective in controlling symptoms in pregnancy (*P* = .021), as seen in Table III. There was no significant association between less adherence and concerns about harm to the baby with asthma rescue therapy (*P* = .436).

**Table III:** Maternal beliefs about asthma medication and reported asthma medication adherence

Belief	Less adherence, no. (%) (n = 40)	More adherence, no. (%) (n = 301)	*P* value
I think that it is unsafe to take my asthma medication during pregnancy			
Agree	11 (27.5%)	26 (8.6%)	.001
Disagree	29 (72.5%)	275 (91.4%)	
I am concerned that my asthma preventive/controller medication will harm my baby			
Agree	21 (52.5%)	84 (27.9%)	.003
Disagree	19 (47.5%)	217 (72.1%)	
I am concerned that my asthma rescue therapy medication will harm my baby			
Agree	15 (39.5%)	89 (31.7%)	.436
Disagree	23 (60.5%)	192 (68.3%)	
My asthma medication is effective in preventing asthma symptoms			
Agree	34 (87.2%)	291 (96.7%)	.021
Disagree	5 (12.8%)	10 (3.3%)	
I do not have enough information about the risks of taking my asthma medication during pregnancy			
Agree	22 (55.0%)	124 (41.2%)	.137
Disagree	18 (45.0%)	177 (58.8%)	
My asthma medication has dangerous side effects during pregnancy			
Agree	8 (20.0%)	43 (14.3%)	.474
Disagree	32 (80.0%)	258 (85.7%)	
With no asthma medication, my asthma symptoms would worsen during pregnancy			
Agree	29 (72.5%)	256 (85.0%)	.074
Disagree	11 (27.5%)	45 (15.0%)	

In the crude or IPTW models, which are described in Table IV, participants with less adherence were 4 times more likely to view asthma medication as unsafe during pregnancy (OR = 4.01 [95% CI = 1.80-8.95]) and nearly 3 times more likely to express concern that preventive/controller medications would harm their baby (OR = 2.86 [95% CI = 1.46-5.58]), as displayed in Table IV. Although participants with poorer adherence were less likely to see asthma medication as effective in preventing symptoms, this association was not seen when adjusted for covariates (OR = 0.23 [95% CI = 0.08-0.72]; aOR = 0.54 [95% CI = 0.20-1.44]). Participants with less adherence had decreased odds of believing that without medication, their asthma symptoms would worsen; this was evident in both the crude and adjusted models (OR = 0.46 [95% CI = 0.22-0.99] and aOR = 0.31 [95% CI = 0.13-0.72]).

**Table IV:** Maternal beliefs about asthma medication and reported asthma medication adherence: Adjusted analysis

Belief	Less adherence, n/N (%)	More adherence, n/N (%)	Crude OR (95% CI)∗	IPTW OR (95% CI)
I think that taking my asthma medication during pregnancy is unsafe	11/40 (27.5)	26/301 (8.6)	4.01 (1.80-8.95)	—†
I am concerned that my preventive/controller asthma medication will harm my baby	21/40 (52.5)	84/301 (27.9)	2.86 (1.46-5.58)	—†
I am concerned that my asthma rescue therapy medication will harm my baby	15/38 (39.5)	89/281 (31.7)	1.41 (0.70-2.83)	1.71 (0.86, 3.43)‡
My asthma medication is effective in preventing asthma symptoms	34/39 (87.2)	291/301 (96.7)	0.23 (0.08-0.72)	0.54 (0.20, 1.44)§
I don't have enough information about the risks of taking my asthma medication during pregnancy	22/40 (55.0)	124/301 (41.2)	1.74 (0.90-3.39)	—†
My asthma medication has dangerous side effects during pregnancy	8/40 (20.0)	43/301 (14.3)	1.50 (0.65-3.47)	—†
With no asthma medication, my asthma symptoms would worsen during pregnancy	29/40 (72.5)	256/301 (85.0)	0.46 (0.22-0.99)	0.31 (0.13, 0.72)‖

∗ Estimated by using logistic regression.

† No covariate selected.

‡ Estimated by using IPTW based on propensity score composed of gestational age at date of survey (categoric).

§ Estimated by using IPTW based on propensity score composed of maternal age, maternal BMI, and gestational age at date of survey (categoric).

‖ Estimated by using IPTW based on propensity score composed of asthma symptom control (mild vs severe).

With respect to outcomes, less adherence was not associated with preterm delivery (OR = 0.54 [95% CI = 0.12-2.38]), SGA from the standpoint of birth weight (aOR = 0.31 [95% CI = 0.06-1.76]), or SGA from the standpoint of birth length (aOR = 0.45 [95% CI = 0.05=4.43]). However, SGA from the standpoint of head circumference (≤10th percentile) was associated with less adherence (aOR = 2.82 [95% CI = 1.08-7.39]) (see Table E1 in the Online Repository at www.jaci-global.org).

In this study, participants’ concern for the safety of asthma medication use during pregnancy aligns with previous research demonstrating apprehension about utilizing asthma medication during pregnancy, particularly among patients who are nonadherent with asthma medication.^8^

However, specific aspects of patients’ concern about asthma medication safety during pregnancy are less well understood. Our findings suggest that perceived risk of asthma medication may vary for different asthma medication classes and that these views may be associated with underlying medication compliance, as participants with less medication adherence were significantly more concerned about the safety of preventive/maintenance inhalers (*P* = .002) but not about the safety of rescue inhalers (*P* = .436). Furthermore, participants with less adherence were nearly 3 times more likely to express concern that preventive/maintenance inhalers would harm their baby (OR = 2.86 [95% CI = 1.46-5.58]). Although the established literature has suggested that pregnant patients significantly overestimated the teratogenicity of oral and inhaled corticosteroids during pregnancy, these views have not been previously examined in the context of reported medication adherence.^9^

In crude analysis, we found that participants with poorer adherence to asthma medication were less likely to agree with the statement that without treatment, their asthma symptoms would worsen in pregnancy (OR = 0.46 [95% CI = 0.22-0.99]), which remained true after adjustment for asthma severity based on asthma symptom control (aOR = 0.31 [95% CI = 0.13-0.72]). Similarly, Murphy et al noted that pregnant asthmatic participants who agreed with the statement “my health at present depends on my asthma medication” were significantly more likely to be adherent with their asthma medication.^8^

We did not find an association between asthma medication adherence and the maternal characteristics that have been reported in previously published literature.^6^ This may have been due to the low prevalence of poor adherence in our sample (11.7%), which contrasts with the findings of previous work indicating that as many as 40% of patients discontinue asthma medications during pregnancy.^6^ The low proportion of reported poor adherence may have been due to a social desirability bias or to the tendency to respond with the perceived “correct” answer (ie, that the participant was adherent). It is also possible that the greater adherence reported in this population may also be due to the specific demographics of participants in this study with higher socioeconomic status than that of the general population and to the fact that they had voluntarily enrolled in an asthma medication study. We acknowledge that the low number of participants reporting poor adherence may bias these findings.

It is well established that uncontrolled asthma is associated with a host of adverse infant outcomes, including preterm delivery and SGA.^4^ However, we did not find an association between less medication adherence and preterm delivery or with SGA from the standpoint of birth weight or length. We found that less adherence was associated with an approximate doubling of risk for smaller head circumference (≤10th percentile aOR = 2.82 [95% CI = 1.08-7.39]). For this infant outcome, missing values may have influenced the finding. To our knowledge, poorly controlled asthma during pregnancy and small infant head circumference have not been associated in prior studies. As with maternal characteristics, it is possible that participants incorrectly reported that they were adherent, which could have resulted in bias toward the null for adverse infant outcomes.

The strengths of this study include the overall sample size for the survey components, availability of data regarding participant characteristics, and insight into participants’ beliefs about medication safety and adherence.

The limitations to this study include lack of diversity in the sample, self-report of medication adherence at a single time point in pregnancy, and methodology that did not allow for more qualitative exploration of beliefs. Most of our participants self-identified as being White (81.8%) and having a high level of education. As uncontrolled asthma disproportionately affects historically marginalized groups, examining beliefs about adherence in a more diverse population is critical. Given the lack of diversity in our participant sample, these results cannot be generalized to the whole population.

However, given that the primary demographic in this study was more educated participants with high socioeconomic status, the finding that participants remained significantly concerned about medication safety is critical for provider awareness. Previous work has established that additional patient education programs can improve ICS adherence and reduce concern for fetal safety.^10^^,^^11^ As pregnant patients often discontinue their asthma medication in the first trimester without physician guidance, preconception counseling and education should be considered.^12^

The findings of this study indicate that significant concern regarding the safety and efficacy of asthma medications during pregnancy, particularly ICSs, exists among this participant population. To comprehensively address participants’ concerns about asthma medication use during pregnancy, a more detailed understanding of the nuanced decision-making process regarding asthma medication use during pregnancy is essential. This study underscores the need for additional research examining the decision-making process regarding medication adherence for pregnant asthmatic women.

**Clinical implications**

Concern regarding the safety and efficacy of asthma medication remains a notable issue among pregnant asthmatic women. Exploring pregnant women’s perceptions of asthma medication remains critical in understanding asthma medication use and adherence during pregnancy.

## Disclosure Statement

Disclosure of potential conflict of interest: The authors declare that they have no relevant conflicts of interest.
