# Supplementary material for: Maternal beliefs and asthma medication adherence during pregnancy
Source: J Allergy Clin Immunol Glob. 2025 Oct 10;5(1):100579. doi: 10.1016/j.jacig.2025.100579 (PMC12597261; doi:10.1016/j.jacig.2025.100579)
Supplement: Supplementary Table [file mmc1.docx]

**Supplementary Table 1:** **Infant Birth Outcomes and Asthma Medication Adherence**

| **Outcome** | **Less Adherence n/N (%)** | **More Adherence n/N (%)** | **Crude OR (CI)^a^** | **IPTW OR (CI)** |
| --- | --- | --- | --- | --- |
| Length ≤10th centile | 1/32 (3.1%) | 10/242 (4.1%) | 0.75 (0.09, 6.05) | 0.45 (0.05, 4.43)^b^ |
| Weight ≤10th centile | 2/32 (6.2%) | 29/253 (11.5%) | 0.51 (0.12, 2.27) | 0.31 (0.06, 1.76)^c^ |
| Occipitofrontal Circumference ≤10th centile | 8/27 (29.6%) | 27/190 (14.2%) | 2.54 (1.01, 6.38) | 2.82 (1.08, 7.39)^d^ |
| Preterm Delivery | 2/33 (6.1%) | 26/243 (10.7%) | 0.54 (0.12, 2.38) | ----^e^ |
| ^a^Estimated using logistic regression. | | | | |
| ^b^Estimated using IPTW based on propensity score composed of asthma symptom control (mild/severe). | | | | |
| ^c^Estimated using IPTW based on propensity score composed of asthma symptom control (mild/severe). | | | | |
| ^d^Estimated using IPTW based on propensity score composed of maternal pre-pregnancy BMI. | | | | |
| ^e^No covariate selected. | | | | |
